# Nutritional intervention for diabetes mellitus with Alzheimer's disease

**DOI:** 10.3389/fnut.2022.1046726

**Published:** 2022-11-15

**Authors:** Zhi Li, Simian Li, Ying Xiao, Tian Zhong, Xi Yu, Ling Wang

**Affiliations:** Faculty of Medicine, Macau University of Science and Technology, Macau, China

**Keywords:** nutritional intervention, dietary patterns, diabetes mellitus, Alzheimer's disease, review

## Abstract

The combined disease burden of diabetes mellitus (DM) and Alzheimer's disease (AD) is increasing, and the two diseases share some common pathological changes. However, the pharmacotherapeutic approach to this clinical complexity is limited to symptomatic rather than disease-arresting, with the possible exception of metformin. Whether nutritional intervention might extend or synergize with these effects of metformin is of interest. In particular, dietary patterns with an emphasis on dietary diversity shown to affect cognitive function are of growing interest in a range of food cultural settings. This paper presents the association between diabetes and AD. In addition, the cross-cultural nutritional intervention programs with the potential to mitigate both insulin resistance (IR) and hyperglycemia, together with cognitive impairment are also reviewed. Both dietary patterns and nutritional supplementation showed the effects of improving glycemic control and reducing cognitive decline in diabetes associated with AD, but the intervention specificity remained controversial. Multi-nutrient supplements combined with diverse diets may have preventive and therapeutic potential for DM combined with AD, at least as related to the B vitamin group and folate-dependent homocysteine (Hcy). The nutritional intervention has promise in the prevention and management of DM and AD comorbidities, and more clinical studies would be of nutritional scientific merit.

## Introduction

Diabetes mellitus (DM) is a chronic disease with a metabolic disorder characterized by hyperglycemia, resulting from insufficient insulin secretion in the body or insulin resistance (IR) (1). According to published data, the global prevalence of diabetes was about 9.3% (463 million people) in 2019, and it is estimated that the prevalence will rise to 10.2% (578 million people) by 2030. More than 700 million individuals might be affected by diabetes worldwide by 2045 (2). At present, the treatment methods for DM mainly include five measures: dietary control, exercise, hypoglycemic medicine, health education, and self-monitoring.

Dementia is also a serious public health problem. Alzheimer's disease (AD), the most common type of dementia, is a chronic neurodegenerative disease characterized by impairment of memory and cognition, along with changes in behavior and personality (3). Pathological features of AD include amyloid plaques composed of fibrillar Aβ and neurofibrillary tangles composed of hyper-phosphorylated tau proteins that could induce oxidative stress, inflammatory damage, cerebrovascular damage, blood-brain barrier dysfunction, and neuronal death (4). The mechanism of AD development is not yet fully discovered and both genetic and environmental factors are involved (5). According to the reports in 2021, AD affected approximately 34 million people worldwide and approximately 5.8 million Americans aged 65 years and older currently have AD. From 2000 to 2019, the number of reported deaths related to AD increased by more than 145% (5, 6). Epidemiological studies have shown that n-3 long-chain polyunsaturated fatty acids (FAs) and docosahexaenoic acid could inhibit Aβ and tau protein production and help the neurons maintain normal function (7). Low intakes of n-3 FAs, B vitamins, and antioxidants have been linked to an increased risk of AD (8, 9). Currently, there is no effective drug that could reverse the symptoms of AD or stop neuronal damage and destruction, and the disease tends to progress gradually.

A substantial body of research indicates that nutrients and phytochemicals including vitamins, folic acid, and polyphenols may have therapeutic effects on DM combined with AD because they could regulate blood glucose concentration, slow the progress of cognitive decline, improve IR, and protect the nervous system from inflammation and/or oxidative damage (10–12). This review discusses and summarizes the effects and potential mechanisms of nutritional intervention of DM and AD, and provides suggestions for nutritional therapy to lower the risk of developing AD in DM individuals.

## Methods

CiteSpace visual analysis software and the bibliometric analysis platform were used to analyze the status of the nutritional intervention that improves DM accompanied with AD. PubMed, EMBASE, Cochrane Library, and China National Knowledge Infrastructure (CNKI) databases were searched to identify the relevant and reliable literature up to June 2022. The following search terms were used: Alzheimer's disease, diabetes, dementia, food ingredients, nutrition, and diet. After screening the abstracts and titles, the relevant literature was analyzed and organized, focusing on the last 5 years. In total, 161 potentially relevant articles were included in this review.

## Correlation between diabetes and Alzheimer's disease

Type 1 diabetes mellitus (T1DM) is a subtype of diabetes (13). Some links have been found between type 1 diabetes and cognitive impairment (14). A study on cerebral compromise and its underlying mechanisms in type 1 diabetes found altered levels of cerebrospinal fluid (CSF), a biomarker of AD, compared to controls. However, the observed profile does not match the full risk profile seen in pre-AD patients (15). The findings of a large retrospective cohort study of 343,062 hospitalized patients with type 1 diabetes indicate that type 1 diabetes is associated with an elevated risk of any dementia, AD, and vascular dementia, and is particularly pronounced in younger patients with diabetes (16). The mechanisms underlying the association between type 1 diabetes and the development of cognitive impairment are not yet fully understood. Further research is needed in the future.

Numerous epidemiological studies have linked diabetes to dementia caused by AD, especially type 2 diabetes mellitus (T2DM), and it is even considered that AD is likely to represent a brain-specific form of diabetes, namely type 3 diabetes. Some studies indicated that T2DM is a risk factor for AD (17), but there is no validation as to whether AD is a risk factor for T2DM. Type 2 diabetes mellitus accounts for one-tenth of people with dementia worldwide, and T2DM patients have a higher chance (>50%) to develop AD than those non-T2DM individuals (18–20). Alzheimer's disease and T2DM share common risk factors, such as obesity, aging, and IR (21–23). In addition, there are many common pathological mechanisms associated with IR between AD and T2DM, such as oxidative stress, impaired insulin signaling, mitochondrial dysfunction, neuro-inflammation, advanced glycation end products (AGEs), and metabolic syndrome, etc. (3, 24, 25). The insulin signaling pathway is initiated when insulin recognizes and binds to the transmembrane tyrosine kinase receptor (TKR). Activated TKR causes insulin receptor substrates (IRS) to be tyrosine phosphorylated by insulin receptor tyrosine kinase, followed by blocking downstream signaling pathways and impairing insulin signaling pathways, then working together with Aβ deposition and mitochondrial dysfunction to form a vicious circle (26).

Abnormalities of glucose metabolism may contribute to AD development in diabetic patients and the disorder of energy metabolism is directly associated with the pathological development of AD (27). Abnormal metabolism and transportation of glucose in DM could impair the cognitive performance of the patients (28). Therefore, adequate blood glucose control should be the main goal of therapeutic approaches to lower the risk of AD in diabetic individuals. Additionally, oxidative stress and mitochondrial dysfunction are linked to the onset and progression of AD in diabetic patients. It is postulated that the normal function of mitochondria is crucial for delaying aging and avoiding neurodegenerative illnesses (29). Mitochondrial dysfunction is a key factor in the pathogenesis of various diseases, such as diabetes and neurodegenerative diseases (30). The brain is very susceptible to mitochondrial dysfunction due to its high energy requirements (31). Many studies have shown that mitochondrial oxidative stress and the production of reactive oxygen species (ROS) are increased under hyperglycemic conditions, and a large amount of ROS could lead to a series of common AD pathological changes, such as the oxidative damage of proteins, carbohydrates, and lipids (32). A recent study found for the first time that adipose tissue-derived EVs from high-fat diet (HFD)-fed mice or patients with diabetes induce remarkable synaptic loss and cognitive impairment (33).

Anti-AD drugs, especially piracetam could significantly reduce some diabetic parameters, such as fasting plasma glucose (FPG), HbA1c, and serum insulin concentration in patients with diabetic AD (34). Similarly, epidemiological studies showed that some antidiabetic drugs such as metformin, applied in diabetic patients could reduce the risk of developing AD and all-cause dementia (4, 35). However, the protective effect of metformin on AD is controversial. One study found that metformin had a protective effect in diabetic patients accompanied by cognitive impairment or dementia, while the protective effect was not seen in non-diabetic individuals with cognitive impairment (36). The patients with mild cognitive impairment or mild AD showed little improvement after being treated with metformin in clinical trials (37, 38). In addition, the precise mechanism underlying the therapeutic activity of metformin in AD is unclear. Metformin may act through mechanisms involving glucose homeostasis, decreased amyloid plaque deposition, normalization of tau protein phosphorylation, and increased autophagy (39). More research is needed to confirm the potential role of metformin in DM accompanied by AD.

## Nutritional intervention improves DM accompanied with AD

Due to the increasing prevalence, mortality, and complication rates, DM has a great impact on the quality of life in diabetic patients. The effect of pharmacology to cure AD is limited, as whether the nutritional intervention could reduce the risk and progression of AD in diabetic individuals is of significant interest. Changes in dietary intake and lifestyle are more important, and easier and safer to implement than medication. It is particularly important to explore scientific and effective nutritional interventions to improve the disease prognosis of DM patients with AD.

### Management of dietary intake

For patients with diabetes and AD, it is common to have both declined cognition and unhealthy dietary intake, overconsumption of some foods makes it difficult to glucose control, while inappropriate strict limitation of food intake might induce hypoglycemia. Management of dietary intake is critical for blood glucose control in diabetic patients. Some studies found that improper dietary control, untimely drug adjustment, and the influence of exercise intensity contributed to poor glucose control in patients with AD accompanied by T2DM (40). Many studies also showed that a reasonable and healthy diet could provide appropriate nutrients and energy required by the patients without inducing significant fluctuation in blood glucose concentration (41–43). Well-planned dietary management could effectively control blood glucose and insulin concentrations and might help to delay further cognitive decline (44) in T2DM patients with AD.

#### Prevention of hypoglycemia

In cases of DM accompanied by dementia or AD, the patients might have a problem organizing their meals and even be insensitive to hungry, which could induce hypoglycemia (45). The caregiver should try his/her best to persuade him/her to eat, be patient with feeding, and if necessary, give a sugar-free liquid diet through tube feeding or provide intravenous nutrition as prescribed by the doctor to meet the needs of the body's energy (46). To prevent hypoglycemia, it is recommended that these patients should bring some candies and chocolates with them. For the in-patients who are not used to adapt the hospital diet, the dietitian should contact their family members to provide the patient's favorite, but low-sugar, low-fat, and fiber-rich foods (47). For those who have difficulty chewing or swallowing, the food with appropriate nutrients and energy should be ground into a paste and provided to the patients (48).

#### Reasonable control of the energy intake

According to the patient's physical activity level and eating habits, the dietitian could formulate a diet guide card, and calculate the daily intake of protein, fat, carbohydrate, vegetable, and fruit based on the total amount of food exchange portion. The dietitian could make an individual diet plan based on the daily calorie requirement of the patient. Coarse grains and pasta are the staple food with a low glycemic index (GI), certain types of dark green leafy vegetables are recommended for diabetic patients even at a relatively large amount, while foods rich in simple sugar, e.g., sucrose and honey should be limited. In general, three meals a day are allocated reasonably at the energy distribution of 2/5, 2/5, and 1/5 for breakfast, lunch, and dinner, respectively (49, 50).

#### Glycemic management

Good glycemic control is crucial for diabetic patients in suppressing disease progress, complications, and even the development of AD. Feeling hungry is a common symptom of DM patients. The quantity and quality of the food consumed should be cautious. Under the condition of enough energy and nutrients, the patients should choose foods that are low GI and rich in dietary fiber, such as coarse grains, potatoes, and miscellaneous beans. Dietary fiber could prevent postprandial hyperglycemia and be fermented by intestinal flora to produce short-chain FAs in the large bowel. Daily dietary fiber intake could reach up to 40 g for diabetic patients. At the same time, the patient's physical activity level and tolerance capacity should be monitored at the time of exercise to avoid hypoglycemia (49).

### Dietary pattern

#### Improving effects of dietary patterns in DM-only patients

Changes in lifestyle and self-management skills are critical in the treatment of diabetes. A change in eating habits is one of the most important lifestyle changes and challenges for people with diabetes (51). Studies have shown that a low-carbohydrate diet (LCD) can improve glycemic control in people with T2DM, and the effects are even more pronounced with a ketogenic diet (KD) (52). However, there is currently no evidence that LCD or KD can delay or prevent the onset of T1DM (53). In addition, a meta-analysis found that Medi (Mediterranean diet) appears to be the most effective and efficient diet for better glycemic control in patients with T2DM (54). There is also evidence that Medi has beneficial effects on cardiometabolic health and reduces mortality in patients with T2DM (55).

#### Improving effects of dietary patterns in AD-only patients

For AD patients, observational studies suggested that the MIND (Mediterranean-DASH Diet Intervention for Neurodegenerative Delay) may be more protective against cognitive decline and AD than the Mediterranean and DASH (Dietary Approaches to Stop Hypertension) diets, but more evidence on the MIND diet is required to make a firm judgment (56–58). Ketogenic diet has beneficial effects on enhancing mitochondrial function and cellular metabolism, which are associated with improved cognitive performance in older adults with AD. The level and duration of ketosis affect the improvement of cognitive outcomes (59).

#### Improving effects of dietary patterns in DM with AD patients

Medi, DASH, and MIND dietary patterns have been found to have a positive influence on DM with AD patients. Both the Medi and DASH dietary patterns emphasize the consumption of plant-based foods and low or limited consumption of red meat. Medi is a traditional diet originating from Mediterranean countries, that focuses on the consumption of abundant fruits, vegetables, legumes, unrefined grains, plenty of fish, and moderate dairy products (such as low-fat cheese and yogurt) and wine, taking olive oil as the cooking oil, while DASH emphasizes consumption of dairy products and low consumption of sodium, industrial sweets, and saturated fat (60). In middle-aged and older adults with T2DM, adherence to the Medi is related to better cognitive function and glycemic control (61, 62). The MIND diet is a combination of the Medi and DASH dietary patterns, emphasizing the consumption of natural plant foods, especially increasing the intake of berries and dark green leafy vegetables, which are rich in folic acid, lutein, lycopene, and alpha and beta carotene, which might play roles in anti-aging for the brain and help to maintain cognitive function (63–65). Adherence to any of the Medi, DASH, and MIND dietary patterns could improve insulin sensitivity, and reduce the likelihood of diabetes and inflammation, in turn reducing the risk of dementia and AD (66–69).

Ketogenic diet has also been found to have a positive influence on DM with AD patients. Ketogenic diets are low-carbohydrate, high-fat, moderate-protein diets that typically provide about 80% of calories from fat, 15% from protein, and 5% from carbohydrates (70). Medium-chain triglyceride (MCT) oil, a major lipid component in KD, may promote ketogenesis and maintain mitochondrial function, in conjunctive therapy for AD patients (71, 72). This dietary pattern could improve glycemic control in T2DM (73) and accompany cognitive impairment in AD (74). Long-term adherence to the KD is difficult with side effects attributable to the compromised dietary quality of micro-nutrient deficiency, along with poor appetite, nausea, constipation, fatigue, dyslipidemia, and unexpected weight loss (53, 75).

### Supplements

Some studies have demonstrated that probiotic supplementation could improve T2DM and AD (76–79). For example, the probiotic *Lactobacillus acidophilus* attenuated the inflammatory response in T2DM by modulating hepatic gluconeogenesis, lipid metabolism, and gut microbiota in mice (80). Prebiotic inulin and dietary inulin may suppress inflammation and modulate gut macrobiotics during T2DM progression (81). In AD patients, cognitive function was improved after treatment with probiotics containing Lactobacillus and Bifidobacterium (82, 83).

It remains debatable whether patients with T2DM and AD could benefit from vitamin supplementation. Vitamin A may be involved in nerve regeneration, neurodevelopment, and neurodegeneration, including AD (84, 85). Vitamin C and E have been touted for potentially favorable anti-oxidative effects in T2DM and AD (86, 87). Vitamin D deficiency might increase neurodegenerative risk, in part through ted development of IR and diabetes by reducing insulin signaling (88, 89). Experimental studies indicated that vitamin D had the potential to prevent and treat cognitive decline in dementia (90), and cohort studies suggested that higher concentrations of circulating vitamin D may lower the risk of all-cause dementia and AD in T2DM. Vitamin D supplementation in the elderly might slow cognitive decline and delay the onset of dementia, especially AD (91, 92).

Hyperhomocysteinemia (HHcy) is a risk factor for AD development, and once HHcy is corrected, the development of AD might be postponed (93–95). Homocysteine (Hcy) is a sulfur-containing non-proteinogenic amino acid that enhances excitotoxicity and causes DNA damage and death in neurons, impairing short-term memory and learning (96). Hyperhomocysteinemia is a state in which the body has an excess of Hcy (97). Vitamins B-6, B-12, and folic acid are essential dietary nutrients for maintaining normal physiological concentrations of Hcy (98). Folic acid (also known as the oxidized form of vitamin B-9) supplementation has positive effects on diabetes-related oxidative stress (99) and could improve cognitive function in AD patients (100). Folic acid supplementation alone or the use of multivitamin supplements containing vitamins B-6, and B-12 have been shown to be effective in reducing plasma Hcy concentrations in patients with AD and DM (101–103). Brain atrophy is accelerated in the elderly with cognitive impairment, particularly those who have AD (104). It has been demonstrated that HHcy is linked to brain atrophy. Supplementation with B vitamins to elderly with mild cognitive impairment may slow the acceleration of brain atrophy by lowering blood Hcy concentrations (105). Additionally, a high baseline of plasma n-3 FA was observed to improve the positive effects of high-dose B vitamin supplementation, which decreased the mean rate of brain shrinkage by 40% in participants with high plasma n-3 FA concentrations compared to those in the placebo group. In contrast, B vitamins had no effect on brain shrinkage in patients with low concentrations of plasma n-3 FA (106). Even though numerous studies explored the association between plasma Hcy and AD, it is not easy to tell the exact effects of Hcy, folate, or vitamin B-12 on cognition and/or AD pathogenesis (107). Understanding the underlying mechanisms of HHcy in AD may help to improve the treatment and bring immediate clinical advantages for the patients.

The antioxidant and anti-inflammatory effects of bioactive compounds, such as polyphenols and carotenoids in vegetables and fruits make them candidates for the prevention and control of DM and AD. Polyphenols such as resveratrol (108–112), gallic acid (11, 113, 114), curcumin (115–118), anthocyanins (119), luteolin (120), hesperetin (121), genistein (122), *Boswellia serrata* gum extract (123), mango leaf extract (124), and flavonoids (125), exist in a variety of plants. They have various functions, such as preventing oxidative stress damage, regulating blood glucose concentration, inhibiting inflammation, improving IR, and neuroprotection, which are all beneficial to DM and AD. A great body of research have confirmed that having foods rich in polyphenols could reduce the risk of DM and AD (12), improve insulin sensitivity in DM patients to inhibit the formation of AGEs, and promote cellular uptake of glucose (126). Polyphenols could also prevent the subsequent development of DM-related complications such as cardiovascular disease, neuropathy, etc., and improve neuronal metabolism and cognitive performance (127), Carotenoids, such as lycopene have been shown having beneficial effects on diabetes and its associated complications in many animal studies, and are potentially effective drug components for the treatment of AD (128, 129).

Supplementation of trace elements, such as magnesium and zinc in diabetic patients could promote glucose transport, maintain normal cell function and lipid metabolism, and enhance tyrosine kinase activity. There was a significant negative association between magnesium intake and all-cause dementia, but the same association was not observed in AD, whereas cognitive improvement was found after zinc therapy in a mouse model of AD, indicating that zinc may play an important role in the pathogenesis of AD (130, 131).

Intake of n-3 FAs (132), pentacyclic triterpenoids (133), Hedera nepalensis crude extract and lupin alcohol (134), marine phenolics (135), fig leaf extract (136), α-lipoic acid (137) has been shown to positively mediate inflammatory and immune responses, reduce the risk of IR and neurocognitive impairment, and ultimately decrease the risk of AD.

The effects of nutritional intervention for DM with AD mentioned above are summarized in Table 1.

**Table 1:** The effects of nutritional intervention for diabetes mellitus with Alzheimer's disease.

**Nutritional intervention**	**Main findings**	**Intervention: features of dietary patterns and supplements**	**Author and Ref. No**.
**Dietary pattern**
Mediterranean diet (Medi)	Reduce the incidence of diabetes	Carbohydrates, % of energy: 39 ± 6 Protein, % of energy: 14 ± 3 Lipids, % of energy: 47 ± 7	Anastasiou et al. (68)
	Improve insulin sensitivity		
Dietary Approaches to Stop High Blood Pressure (DASH)	Reduce oxidative stress and inflammation	Carbohydrate, % of energy: 55 ± 2 Protein, % of energy: 16 ± 0.9 Total fat, % of energy: 29 ± 1.1 Sodium intake: 2,400 mg/d	Azadbakht et al. (67)
Mediterranean-DASH Delayed Intervention for Neurodegeneration (MIND)	Prevent dementia and reduce the risk of AD	Carbohydrates: 45–55% Protein: 15–20% Total fat: 25–30% Sodium intake: 2,400 mg/d	Asemi et al. (69)
Ketogenic diets (KD)	Control and improve blood glucose in patients with T2DM	14% Carbohydrates (< 50 g) 28% Protein 58% Fat (35% MUFA, 13% PUFA, < 10% SFA)	Brinkworth et al. (73)
	Improve cognition in AD patients	58% Fat (26% SFA, 32% non-saturated) 29% Protein 7% Fiber 6% Carbohydrates	Abboud et al. (74)
**Supplements**	
Probiotic *Lactobacillus acidophilus*	Improve the intestinal barrier function	Untreated diabetes mellitus group (DC), diabetic mice treated with *L. acidophilus* KLDS1.1003 (LA1, 1 × 109 CFU/d) and diabetic mice treated with *L. acidophilus* KLDS1.0901 group (LA2, 1 × 109 CFU/d).	Yan et al. (79)
	Suppress inflammatory responses in liver and colon		
	Regulate glucose and lipid metabolism in liver		
Dietary inulin	Reduce the plasma LPS concentration	Six groups (15 mice per group): pre-diabetic group (PDM group); inulin-treated pre-diabetic group (INU/PDM group); early diabetic group (EDM group); inulin-treated early diabetic group (INU/EDM group); diabetic group (DM group); inulin-treated diabetic group (INU/DM group).	Li et al. (81)
	Alter plasma pro-inflammatory and anti-inflammatory cytokines in diverse stages of T2DM		
	Modulate gut dysbiosis in diverse stages of T2DM		
Probiotics containing Lactobacillus and Bifido-bacterium	Decrease the plasma MDA and the serum hs-CRP concentrations	Control group: treat with milk. Probiotic group: take 200 ml/day probiotic milk containing *Lactobacillus acidophilus, Lactobacillus casei, Bifidobacterium bifidum*, and *Lactobacillus fermentum* (2 × 109 CFU/g for each) for 12 weeks.	Akbari et al. (82)
	Reduce the HOMA-B index		
	Decrease the serum triglyceride and VLDL concentrations		
Vitamin A	Adequate intake of vitamin A may help protecting against diabetes, especially for men	Vitamin A intakes were assessed by three consecutive 24-h dietary recalls combined with a household food inventory.	Su et al. (84)
	Modulate Aβ formation and aggregation, cholinergic transmission, and inflammatory responses	500 IU (control diet)/100 g of VitA or 2,000 IU (enriched diet)/100 g VitA.	Biyong et al. (85)
Vitamin B	Slow the acceleration of brain atrophy	All patients received treatment with an acetylcholinesterase inhibitor and were randomized to receive add-on mecobalamin (B12) 500 mg + multivitamin supplement, or placebos. (Multivitamin: pyridoxine (B6) 5 mg, folic acid 1 mg, and other vitamins and iron).	Sun et al. (101)
	Reduce plasma homocysteine concentrations in patients with Alzheimer's disease and diabetes		
	Improve cognitive function in AD patients		
Vitamin C	Avoid the pro-oxidative effect	VC supplementation of 500–1,000 mg/day.	Sun et al. (86)
	Reduce the risk of diabetes mellitus type 2 complications		
	A reduced risk of cognitive decline	Exposure to supplements of vitamin E or C was measured at baseline using data collected either from the structured interview at the clinical examination or the selfadministered risk factor questionnaire (RFQ).	Basambombo et al. (87)
Vitamin D	Improve adipocyte metabolic function and protect against obesity	Vitamin D 1,25-dihydroxyvitamin D[1,25(OH)2D] treatment (24 h, 100 nmol/L).	Chang et al. (88)
	Reduce low-grade chronic inflammation caused by IR	2,000 IU of Vitamin D/day.	Wenclewska et al. (89)
	Preserve brain health and lower risk of AD	Plasma 25(OH)D concentrations: ≥50 nmol/L.	Feart et al. (91)
	Slow cognitive decline	Serum 25(OH)D concentrations: ≥50 nmol/L.	Geng et al. (92)
	Delay or prevent the onset of dementia, especially AD		
Polyphenols	Insulin resistance ↓	Resveratrol dose: 500 mg twice a day 45 days.	Movahed et al. (112)
	Blood glucose levels ↓		
	HOMA-β ↓		
	Systolic blood pressure ↓		
	Neuroprotection	—	Ben Hmidene et al. (125)
Trace elements (magnesium and zinc)	Lower serum cholesterol and cholesterol/high-density lipoprotein ratio	ZnSO_4_: 22 mg/day.	Gunasekara et al. (130)
	Maintain normal cell function and normal lipid metabolism	Mg supplementation: 4.5 g/day of Mg pidolate (368 mg/day of Mg ion).	Barbagallo et al. (131)
	Enhance tyrosine kinase activity		
n-3 Fatty acids (FAs)	Mediate inflammatory and immune responses	Omega-3 fatty acid diet (n-3 diet): add 0.5% of flaxseed oil and 1.2% of docosahexaenoic acid capsule oil.	Agrawal et al. (132)
	Reduce the risk of IR and neurocognitive impairment		
Pentacyclic triterpenoids	Inhibit TNF-α and TGF-β production	0.1% FCS and different concentrations of betulin (1 or 10 μM) or betulinic acid (1 or 5 μM) (Sigma–Aldrich).	Szuster-Ciesielska et al. (133)
	Exert an antifibrotic activity by silencing ethanol-activated HSCs		
Hedera nepalensis crude extract (HNC) and lupin alcohol	HNC and lupeol ameliorate the increase in plasma glucose level and enhance the antioxidant enzymes profile	NC (normal control) group (non-diabetic, non-Alzheimer): given 10% DMSO (10 ml/kg BW/day). STZ + AlCl_3_ co-treated group: received Rivastigmine (EXCELON^®^) at 0.8 mg/kg BW/day as positive control (PC). AC group (Alzheimer control): received 10% DMSO (10 ml/kg BW/day) after STZ + AlCl_3_ co-treatment. HNC group (AC + HNC): received crude extract 400 mg/kg (BW) after STZ + AlCl_3_ co-treatment. AC + Lupeol group: received lupeol orally at 10 mg/kg (BW) after STZ + AlCl3 co-treatment.	Hashmi et al. (134)
	Decrease DM related complications of AD such as cognitive impairment and anxiety/fear related behavioral indices		
Marine phenolics	Decrease postprandial glucose, insulin, and C-peptide levels	Dieckol-rich extract: 1,500 mg/day.	Lee et al. (135)
Fig leaf extract	The methanol and water extracts of *Ficus carica* leaf extracts have strong antioxidant activity	First 150 μl of the extract was mixed with 50 μl of 1.0 × 10^−3^ M freshly prepared DPPH• methanol solution in 96-well plates.	Ergül et al. (136)
	Show strong α-glucosidase and α-amylase inhibition activity		
α-lipoic acid (ALA)	Show the improvement in insulin sensitivity and lipid metabolism	ALA: 300 mg/daily for 3 months.	Udupa et al. (137)

## Conclusion

The prevalence of DM and AD increases year by year. These two diseases share a common underlying pathological mechanism, and DM patients have a higher risk of developing AD. Therefore, a focus on how to prevent and treat DM accompanied by AD should underscore the potential relevance of nutritional intervention strategies in three respects: food intake management, dietary pattern, and nutrient supplementation. To date, various food factors and dietary components including probiotics, polyphenols, trace elements, and n-3 FAs, have been countenanced as candidates for cognitive maintenance or improvement in diabetic patients who are prone to brain dysfunction. Two dietary patterns, the KD, and the Mediterranean diet have been found to be therapeutically effective in DM and AD. Multi-nutrient supplements together with a healthy dietary pattern may enhance the therapeutic effectiveness. The key to the prevention of DM accompanied by AD is to identify the risk factors such as obesity, lack of exercise, and unhealthy eating habits before the disease is apparent, and to establish effective intervention strategies, especially nutritional measures.

## Author contributions

ZL: conducted literature search in different search engines, contributed in preparation of first draft, and final version of the manuscript. SL: conducted literature search in different search engines and contributed in preparation of first draft of the manuscript. YX, TZ, and XY: provided guidance of the manuscript. LW: critically reviewed the manuscript, provided guidance, and approved the final version of the manuscript. All authors contributed to the article and approved the submitted version.

## Funding

The work is supported by Macau University of Science and Technology, Faculty Research Grant, FRG-21-036-FMD.

## Conflict of interest

The authors declare that the research was conducted in the absence of any commercial or financial relationships that could be construed as a potential conflict of interest.

## Publisher's note

All claims expressed in this article are solely those of the authors and do not necessarily represent those of their affiliated organizations, or those of the publisher, the editors and the reviewers. Any product that may be evaluated in this article, or claim that may be made by its manufacturer, is not guaranteed or endorsed by the publisher.
